# Resistance of PETG Materials on Thermocycling and Brushing

**DOI:** 10.3390/dj11050135

**Published:** 2023-05-16

**Authors:** Luka Šimunović, Tadeja Blagec, Senka Meštrović

**Affiliations:** Department of Orthodontics, School of Dental Medicine Zagreb, University of Zagreb, 10000 Zagreb, Croatia; lsimunovic@sfzg.hr (L.Š.); tblagec@sfzg.hr (T.B.)

**Keywords:** thermocycling, brushing, retainers, PETG

## Abstract

The aim was to assess the impact of thermocycling and brushing on the surface roughness and mass of PETG material—the most commonly used for orthodontic retainers. A total of 96 specimens were exposed to thermocycling and brushing with three different kinds of toothbrushes depending on the number and thickness of the bristles. Surface roughness and mass were evaluated three times: initially, after thermocycling, and after brushing. In all four brands, both thermocycling and brushing increased surface roughness significantly (*p* < 0.001), with Biolon having the lowest and Track A having the highest. In terms of brushing, only Biolon samples showed statistically significant increased roughness after brushing with all three types of brushes, in comparison to Erkodur A1, where differences were not statistically significant. Thermocycling increased the mass of all samples, but a statistically significant difference was found only in Biolon (*p* = 0.0203), while after brushing, decreased mass was found in all specimens, statistically significant only in Essix C+ (CS 1560: *p* = 0.016). PETG material showed instability when exposed to external influences- thermocycling produced an increase in roughness and mass, and brushing mostly caused an increase in roughness and decrease in mass. Erkodur A1 demonstrated the greatest stability, whereas Biolon demonstrated the lowest.

## 1. Introduction

Retention and dental arch form maintenance after active orthodontic treatment is a very challenging part of the orthodontic specialty. There are several types of retainers available on the market, and it is up to the orthodontic clinician to decide which one is most suitable for the situation.

In 1971, Ponitz [1] introduced a new type of removable retainer made of thermoplastic material, which represented an alternative to the traditional Hawley removable retainer. However, the use of it gained popularity after 1993 when Sheridan [2] proposed Essix retainers made of translucent acrylic, which was heated and vacuum or pressure-formed over the study cast; it was also reported that this material was easy to clean, esthetic, and cost about one-third less than the Hawley retainer [1,3].

Thermoplastic orthodontic retainers are becoming more popular each day. Low hardness, transparency, aging resistance, high elasticity, and resilience are desirable qualities of these orthodontic appliances [4]. There are various materials on the market used for their manufacturing: polyurethane, polypropylene, polyethylene, polyethylene terephthalate, and polyethylene terephthalate glycol [5,6]. PET (polyethylene terephthalate) is a polymer that is frequently utilized in daily life. This material is used to create items such as food containers, drink bottles, films, and fibers. Its widespread use is due to the crystallization process, which raises a material’s density, stability, gas permeability, and strength [7].

The PET-G (polyethylene glycol-co-cyclohexane-1,4-dimethanol terephthalate) is an amorphous copolymer of PET with the additional glycol group [8,9]. This glycol molecule addition reduces the crystallinity level of the material by disrupting the order of the polymer chain, thus making the material more suitable for printing since, nowadays, PETG has become a material of choice for 3D printing [10,11]. *PETG* material, on the other hand, is less common in industry than PET material since it cannot crystallize [7]. However, the mechanical properties of the PETG copolymer are close to those of PET [11].

It is synthesized by the polycondensation of 1,4-cyclohexanedimethanol, ethylene glycol, and terephthalic acid in certain proportions [8,9]. It has good mechanical properties, thermoformability, high chemical resistance, excellent transparency, high ductility and chemical resistance, processability, and recyclability [12,13,14,15]. Moreover, it is biodegradable and biocompatible, which makes him suitable for tissue engineering [13]. Furthermore, it shows good fluidity, better transparency, and strong solvent resistance [3]. Due to those great features, it is not only used in medicine but also in many other industries and sectors [13]. Nowadays, blending current thermoplastic materials is an effective way to modify the present and to create new materials with outstanding mechanical properties, low water absorption, great strength, and dimensional stability [3]. All of the aforementioned materials can be blended in different ratios to produce thermoplastic material of higher quality. Even though most polymer blends and composites have their properties tailored to attain a stiffness–ductility balance, this is still a difficult problem [9].

Moreover, manufacturing of thermoplastic retainers is simple and low cost. Even auxiliary personnel with only minimal instruction and experience can fabricate these retainers. No knowledge about acrylic material or technical proficiency in wire bending is necessary [3].

Thermoplastic retainers are easily accepted by patients due to their simplicity of application and esthetic appearance because they are made of versatile transparent polymers [16,17,18,19]. Their molecular, biochemical, morphological, and mechanical properties can be impaired by various influences present in the intraoral environment [20]. Even though they are widely in use, their surface and dimensional stability have not yet been well explored. Jin et al. [21] found that their long-term survival rate is less than one year in comparison to fixed and Hawley retainers, in which the median survival time was more than four years. Surface roughness is a measurement of micro-irregularities on the surface structure [22]. This property of thermoplastic material is determined not only by the characteristics of each material but also by the thermoforming parameters and oral environmental conditions [23,24,25]. Increased surface roughness may result in increased bacterial adhesion, patient discomfort, pigment accumulation, and other unfavorable outcomes [26,27,28]. Retainers must tightly fit over teeth to maintain stability after orthodontic treatment. Therefore, any dimensional changes, mass or volume increase or decrease, need to be avoided or at least reduced to a minimum.

Patients are advised to maintain the hygiene of their retainers. The cleaning of retainers is of great importance in terms of oral and systemic health [29]. Their presence in the oral cavity affects the oral flora with an increase in the cariogenic bacteria Streptococcus mutans (SM) and Lactobacillus (LB) [29,30]. There are different cleaning methods proposed for the patients. Thus, there are no uniform guidelines for the hygiene maintenance of thermoplastic orthodontic retainers. According to one study, cleaning with a toothbrush is recommended in 99.8% of cases [31]. Several studies investigated how brushing with a toothbrush with or without dentifrice affects the surface roughness of thermoplastic retainers [23,32,33,34]. However, the results are not uniform. Moreover, it must be remembered that brushing could scratch the retainer’s surface, creating more area that is favorable for bacterial colonization.

There are no studies addressing the impact of brushing on the mass changes of thermoplastic materials at the moment. It has been proved that thermocycling can also increase the surface roughness of various dental materials [24,35]. Ihssen et al. [36] investigated how thermocycling-accelerated aging impacts the mechanical characteristics of PETG material, but no research has yet been done on how it affects the surface roughness of any thermoplastic material. Only one research examined how thermocycling affected PETG weight change. The results revealed that water absorption causes a statistically significant increase in the mass of thermoplastic specimens [36]. There is currently insufficient data on how external factors affect the surface roughness and mass of vacuum-formed orthodontic retainers. The purpose of this study was to assess the impact of thermocycling and brushing on the surface roughness and mass of PETG specimens.

The null hypothesis of the study is that brushing and thermocycling do not affect the surface roughness and mass of thermoplastic PETG material.

## 2. Materials and Methods

### 2.1. Specimen Preparation

For the purpose of this study, ninety-six specimens were prepared from polyethylene terephthalate glycol (PETG), the most commonly used material for orthodontic retainers. PETG material from four different manufacturers was evaluated in this study: Erkodur-A1 (Erkodent, Erich Kopp GmbH, Pfalzgrafenweiler, Germany), Biolon (Dreve, Unna, Germany), Track A (Forestadent, Bernhard Fӧrster GmbH, Pforzheim, Germany) and Essix C+ (Raintree Essix, New Orleans, LA, USA). The thickness of the material was 1 mm. Models were prepared according to the protocol of the previous study [32]. A total of 24 samples of each brand were investigated.

### 2.2. Thermocycling

The maximum number of thermocycles (50 cycles) that could be acquired from the literature to simulate one day’s wear was used [37]. A common procedure in dentistry materials science, ISO Norm TS 11405:2015, served as the foundation for the thermocycling regimen [38]. High reproducibility and methodical conformity were thus guaranteed. To simulate wearing the retainer for 30 days, the specimens must be submerged in purified water for 24 h before being thermocycled 1500 times at 5–55 °C 20 s each.

### 2.3. Brushing

Simulated brushing was performed on a self-made device modeled after SD Mechatronik Germany. Depending on the number and thickness of bristles, three different kinds of toothbrushes were used to brush the samples (Curaprox CS 5460, CS 3960, CS 1560; Curaden AG, Kriens, Switzerland). Each type of toothbrush was used for eight samples of each thermoplastic material brand. To mimic 30 days of cumulative brushing, each specimen was brushed for a total of 30 min–15 min on each side (top and bottom) (30 s per day). The cleaning force was set to 2 N, and the cleaning movement was set at 3 cm longitudinally at a rate of 120 strokes per minute [39].

### 2.4. Evaluation of Surface Roughness and Mass

The surface roughness of each specimen was measured with a high-precision profilometer (Mitutoyo SJ-210 surface roughness tester, Mitutoyo, Japan), according to the ISO 4287:1997 standard [40] at the center of the specimens. The roughness parameter Ra was taken into account since it represents the mean arithmetic deviation of the profile; on the unit length of the surface of the total amount of roughness amplitudes, the mean value was calculated. The mass/weight of the samples was measured with the high-precision weight scale (Mettler–Toledo).

Surface roughness and mass were evaluated three times: initially, after thermocycling, and after brushing. After thermocycling, each specimen was dried with a paper towel to ensure accurate mass and roughness measurements.

The study was approved by the Ethic committee of the School of Dental Medicine, University of Zagreb (approval number: 05-PA-30-14-1/2023).

### 2.5. Statistical Analysis

An analysis of data normality using the Shapiro–Wilk test and asymmetry tests revealed a non-normal distribution of initial surface roughness and mass values. Considering there was no difference in top side initial surface roughness values between brand groups, Wilcoxon matched pairs test was applied for intragroup comparison (initial—after thermocycling and after thermocycling—after brushing), while for intergroup comparison Kruskal–Wallis test with post hoc Dunn’s test for multiple p comparison. Bottom side specimens showed statistically different values in initial measurement, so the effect of thermocycling and brushing was presented as a change. One-way ANOVA was utilized for analysis with the post-hoc Tukey HSD test since, according to the Leaven test, the assumption of homogeneity of variances was met. *p* < 0.05 was considered significant.

## 3. Results

The initial surface roughness values of the top side did not vary significantly between the studied brands. Track A had the lowest values, while Essix C+ had the greatest (Table S1.). Essix C+ and Track A had significantly higher initial mass values than Biolon and Erkodur A1 (*p* = 0.01). The highest mass was found in Essix C+, while the lowest was in Erkodur A1 samples.

### 3.1. Effect of Thermocycling on the Top Side

**Figure 1:**
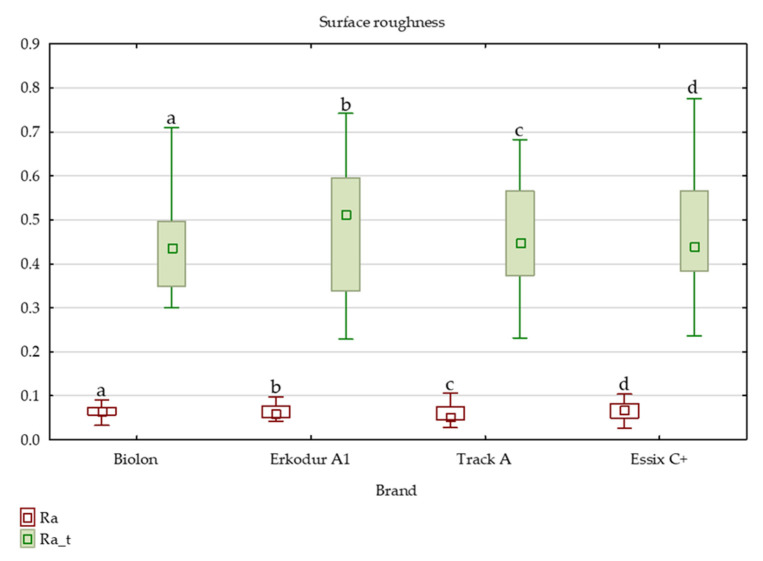
Surface roughness of top sides of specimens after thermocycling. Variables noted with the same letter show statistically significant differences. Ra presents initial Ra values, Ra_t presents surface roughness values after thermocycling.

In all brands, thermocycling substantially increased surface roughness (*p* < 0.01). The effect of thermocycling on surface roughness was highest in Erkodur A1 and least in Biolon (Figure 1. and Table S2). Surface roughness values after thermocycling did not vary significantly between brands, with Biolon having the lowest and Track A having the highest.

Thermocycling increased the mass of all brands tested, but only Biolon showed a statistically significant rise (*p* = 0.0203).

### 3.2. Effect of Brushing on the Top Side

In all brands, brushing led to a statistically significant increase in surface roughness (*p* < 0.01) (Table S2).

According to the number and thickness of the filaments, a statistically significant difference was found between ultra-soft (CS 5460) and soft toothbrushes (CS 1560) in all samples (*p* = 0.0006). Within brand group analysis revealed statistically significant differences in surface roughness between ultra-soft (CS 5460) and soft (CS 1560) toothbrushes in Biolon (*p* = 0.0043) and Essix C+ (*p* = 0.021) samples. In Erkodur A1 and Track A specimens, differences were insignificant.

Surface roughness significantly increased in Biolon samples after brushing with all three types of toothbrushes compared to values after thermocycling (CS 5460: *p* = 0.046; CS 3960: *p* = 0.027; CS 1560: *p* = 0.027), while in Erkodur A1 samples, the difference was not statistically significant.

**Figure 2:**
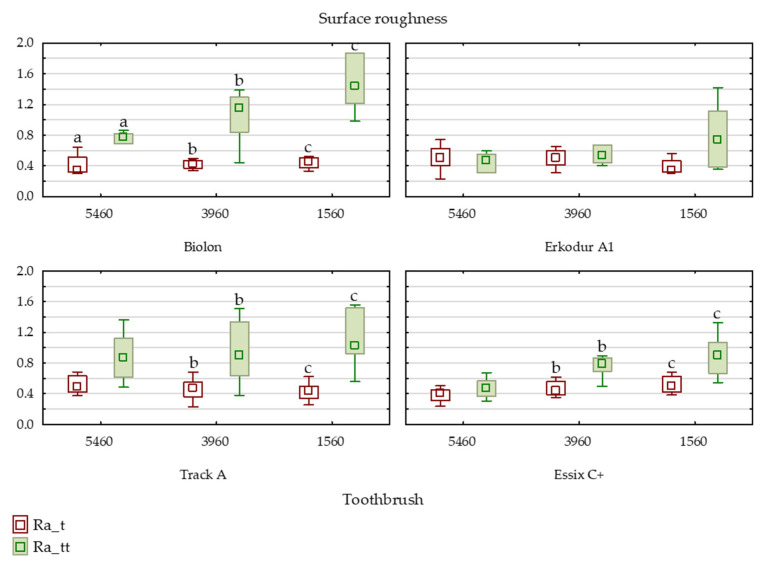
Surface roughness of top sides of specimens after brushing simulation with three different toothbrush types. Variables noted with the same letter show statistically significant differences. Ra_t presents Ra values after thermocycling, Ra_tt presents Ra values after thermocycling and brushing.

Super soft (CS 3960) and soft (CS 1560) toothbrushes significantly increased roughness in Track A and Essix C+ samples (Figure 2).

Brushing caused a decrease in mass in all examined specimens (*p* = 0.0124). Only the CS 1560 toothbrush caused a significant decrease (*p* = 0.028) in Essix C+ specimens.

### 3.3. Effect of Thermocycling on the Bottom Side

Erkodur A1 and Track A had the greatest and lowest initial surface roughness values for the bottom side, respectively. Change in surface roughness in Track A was statistically significantly higher from all other brands (Table S3).

**Figure 3:**
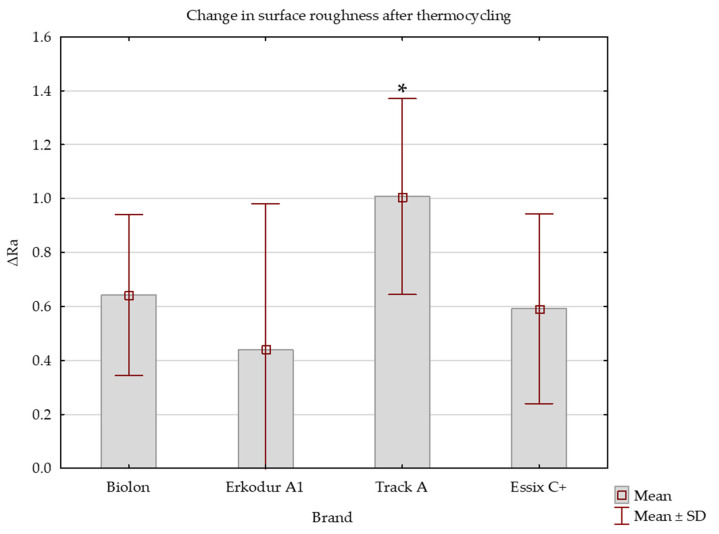
Change in surface roughness of bottom sides of specimens after thermocycling. * statistically significant difference in surface roughness change in comparison to other brand groups.

In all brands, thermocycling led to a statistically significant increase in surface roughness (*p* < 0.01). The effect was highest in Track A and lowest in Erkodur A1 (Figure 3).

### 3.4. Effect of Brushing on the Bottom Side

In all brands, brushing caused a statistically significant increase in surface roughness (*p* = 0.0023) (Table S3). In all samples, there was no significant growth of Ra values when samples were brushed with ultra (CS 5460) and super soft toothbrushes (CS 3960).

**Figure 4:**
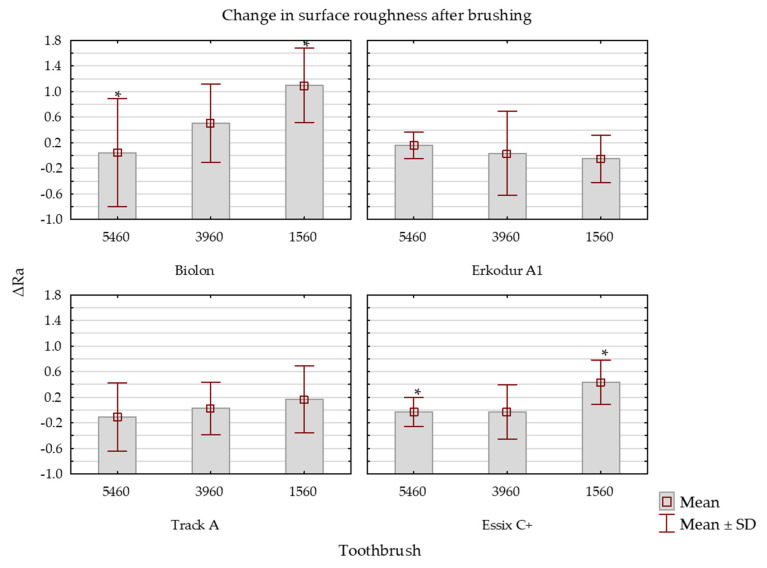
Change in surface roughness of bottom sides of specimens after brushing simulation with three different toothbrush types. * the marked groups show statistically significant differences in surface roughness change.

In Biolon and Essix C+ samples, surface roughness increased significantly after they were exposed to a soft brush in comparison to an ultra-soft brush (CS 1560; *p* = 0.028; *p* = 0.03; Figure 4).

## 4. Discussion

The present study evaluated the influence of thermocycling and brushing on orthodontic retainer materials. As PETG material is one of the most used for their fabrication [37,41], we chose it for this study. PETG is a non-crystalline amorphous polymer that shows excellent mechanical, esthetic, and chemical properties [20]. It is well-known that thermoplastic orthodontic retainers are highly viscoelastic materials susceptible to humidity and temperature changes [42].

Thermocycling is a procedure used to simulate extreme oral environment conditions such as excessive temperature changes and high humidity for a certain period [43]. The mechanical properties of the material exposed to thermocycling are subjected not only to extreme temperature changes but also to water absorption [36,43], which can change the mechanical properties of thermoplastic material, and according to the literature, PETG is among the materials that show the highest water absorption values [20,36,44,45]. When the water absorption is higher, the degradation of the material will be more evident [40]. Furthermore, it has been shown that higher temperature enables higher diffusion of water molecules inside the material. Hence, the higher the intraoral temperature, the greater the water absorption [40]. Apart from the great humidity, salivary enzymes, and continuous and intermittent forces, retainers are also subjected to temperature variations [45]. The temperature in the oral cavity can reach 57 °C after the consumption of hot beverages or foods, and it may take several minutes to return to its original values [45]. These temperature changes can also affect the mechanical properties of the thermoplastic material [44,45,46,47,48].

Several studies investigated the influence of thermal cycling on the different mechanical properties of PETG thermoplastic materials [36,47], but there is a lack of those that deal with the influence of thermocycling on surface roughness. In this study, thermocycling led to a statistically significant increase in surface roughness in all tested brands. Hence, each material had a significantly rougher surface after thermocycling. An explanation for this could be high water absorption causing hydrolytic degradation, which, according to the literature, in the end, results in increased Ra values [24,35]. Daniele et al. [41] proved that PETG material, after immersion in water for 14 days, shows an increase in surface irregularities and amount of impurities, which tend to worsen as the temperature of the water rises. That corresponds with our findings. The results from this study also indicated that surface roughness values after thermocycling did not vary significantly among brands. Although the greatest change of Ra values was observed on the top side of Erkodur A1 specimens, final values were still lower than in other brands, even though this brand initially had a smoother surface (lower Ra values) than others. Hence, thermocycling had the greatest influence on the top side of Erkodur A1. Biolon samples had the least variation in roughness. The impact of thermocycling on the bottom side was greatest in Track A and least in Erkodur A1. This could be explained by the difference in the structure of PETG materials.

Another evaluated stability parameter in our study was the mass/weight of the specimens. The weight of an object or material is the most relevant value to explain water absorption [49]. Of course, water absorption during thermocycling led to an increase in the mass of all tested brands, but only Biolon showed a statistically significant rise. In Moreno Nieto et al. [49] study, tested PETG specimens presented a weight variation rate of 0.3% after exposure to water, while in our study, the mass increased by 0.5%. However, it is important to mention that the results can not be completely compared with our findings since their PETG material specimens were printed from recycled material. Ihssen et al. [36] also investigated mass changes of the PETG specimens after exposure to thermocycling and presented a statistically significant mean relative weight increase of 0.39%. Moreover, the weight of specimens exposed to thermocycling was 48% greater than specimens immersed in water. In this case, probably temperature rise during the thermocycling procedure caused greater absorbance of water. When a material is in a humid environment, water chemically reacts with the polymer matrix in a process called hydrolysis. This consequently leads to the hydrolytic degradation of the material and also swelling phenomena [23,40]. Furthermore, water penetrates the structure of the polymer and acts as a spacer between chains and results in hygroscopic expansion [7,40]. All aforementioned leads to increased volume and weight of the thermoplastic specimens [49]. According to previous studies, water penetrates the amorphous regions of polymers, while crystalline parts of the material remain unaffected [23]. Some studies claim that PETG behaves more stably than other materials in a humid environment due to its high degree of crystallinity [49]. Zhang et al. [50] claim that modified blend PETG/PC/TPU material has a lower water absorption rate than PETG material itself.

In the present study, samples from each one of the four brands were exposed to simulated brushing. There are several ways of maintaining the hygiene of removable retainers, but mechanical cleaning with a toothbrush is the most recommended method, as it is simple and cost-effective [31,51,52]. That is why this method of cleaning was chosen in the present study. Specimens were brushed with three types of toothbrushes according to the number and thickness of the filaments. These filaments/bristles are made of polyester, and they are harder and absorb water six times slower than the nylon bristles, which enables them to keep their original texture even when wet. The softest (ultra soft) brush had 5460 filaments with a thickness of 0.1 mm (CS 5460). Moreover, a super soft brush with 3960 filaments and a thickness of 0.12 mm (CS 3960) and a soft brush with 1560 filaments and 0.15 mm thick (CS 1560) were used in this study. Thicker filaments are stiffer than thinner ones. It can be concluded that with the increasing bristle stiffness, the rubbing and abrasive effect of a toothbrush also increases [53]. A statistically significant difference was found between ultra-soft (CS 5460) and soft toothbrushes (CS 1560) in all samples. Harder toothbrushes created greater surface irregularities of PETG material than softer ones. Stiffer bristles probably created greater scratches, cavities, and irregularities on the specimen’s surface. Currently, there are no studies that compare the influence of toothbrushes with different softness on the surface quality of thermoplastic material. Surface roughness of the top side of specimens significantly increased in Biolon samples after brushing with all three types of toothbrushes, in contrast to Erkodur A1 samples, where the difference was not statistically significant. Therefore, Erkodur A1 samples showed the greatest stability. In Biolon samples, even the softest (CS 5460) toothbrush significantly influenced surface morphology. Even though these tested samples are all made from the same material, different results may be explained by the difference in the manufacturing process and the structure of the material. However, in the Porojan et al. [23] study, Biolon material showed the most constant behavior in terms of surface roughness after being exposed to external influences. These results can not be completely compared with ours since different brands of PETG materials were used. Moreover, super soft (CS 3960) and soft (CS 1560) toothbrushes greatly increased roughness on the top side of Track A and Essix C+ samples. With regard to the bottom side of the specimens, in all brands, there was no significant growth of Ra values when brushed with ultra (CS 5460) and super soft toothbrushes (CS 3960). Only the stiffest toothbrush caused a significant increase in surface roughness in Biolon and Essix C+ samples. Therefore, brushing simulation had a lower influence on the bottom than on the top side of the samples. Since the bottom side initially had greater values of surface roughness, it can be assumed that brushing had a smaller influence on already present greater surface irregularities. In contrast to our study, Wible et al. [33] did not find a difference in surface roughness after copolyester (Essix ACE) material was exposed to brushing. Initial and final Ra values were below 0.2 μm, which is also contradictory with our results. However, the results can not be completely compared with our findings due to differences in methodology and different investigated materials.

The final part of our examination was to evaluate the effect of brushing on the mass of the PETG specimens. According to our results, brushing decreased mass in all specimens, but the difference was statistically significant only in Essix C+ samples with the stiffest toothbrush (CS 1560). This weight reduction of the specimens after brushing may be the result of the removal of the absorbed water particles adhering to the surface of the sample [49]. According to our results, stiffer toothbrushes cause a greater mass reduction.

Final surface roughness values after exposure of PETG material to thermocycling and brushing were all above 0.2 μm, which is clinically relevant in terms of bacterial adhesion [26,54,55]. Furthermore, values above 0.5 μm can cause patient discomfort [56]. Moreover, rougher surfaces might result in undesirable aesthetic effects due to pigment accumulation and loss of transparency [28,33,34]. An increase in the mass or volume of a thermoplastic retainer can lead to unfitting and, consequently, failure to maintain teeth alignment after orthodontic therapy.

In the present study, thermocycling and brushing simulated the wear of the material for 30 days. This could be a limitation of this study. It would be more accurate to simulate a period of one or even a few years because removable retainers are usually replaced every two to three years. Future studies are needed to investigate the duration of the thermoplastic retainer and the period when the worn-out retainer needs to be replaced with the new one. Another limitation is that we only observed PETG, and the stability of other orthodontic retainer materials was not evaluated. In future studies, other thermoplastic materials should also be assessed. Moreover, the flat shape of our specimens did not completely reflect the configuration of the real retainer. Furthermore, investigation of other mechanical and stability properties of orthodontic retainer material is recommended. In vivo studies would be more accurate.

## 5. Conclusions

PETG material showed instability when exposed to external influences- thermocycling produced an increase in roughness and mass, and brushing mostly caused an increase in roughness and decrease in mass. Of all brands, Erkodur A1 demonstrated the greatest stability under external influence, whereas Biolon demonstrated the lowest. Due to the results of this study, it should be proposed to our patients that retainers, when they are not in use, need to be kept on some dry surface/place, away from the water and humid environment. Moreover, hot drinks and food need to be avoided. Removable retainers should be brushed with an ultra-soft brush in order to maintain the surface stability of the material.

## Data Availability

The data presented in this study are available on request from the corresponding author.

## Supplementary Materials

The following supporting information can be downloaded at: https://www.mdpi.com/article/10.3390/dj11050135/s1, Table S1: Initial values of surface roughness (Ra); Table S2: Descriptive statistics of surface roughness values (Ra) after thermocycling and brushing with three different toothbrushes on the top side of specimens; Table S3: Descriptive statistics of surface roughness change (ΔRa) after thermocycling and brushing with three different toothbrushes on the bottom side of specimens.
