# Supplementary material for: Resistance of PETG Materials on Thermocycling and Brushing
Source: Dent J (Basel). 2023 May 16;11(5):135. doi: 10.3390/dj11050135 (PMC10216975; doi:10.3390/dj11050135)
Supplement: Supplementary file 1 [file dentistry-11-00135-s001.zip › dentistry-2319634-supplementary.pdf]

Suppl. Table S1. Initial values of surface roughness (Ra)

Brand	Side	MEDIAN	Q1	Q3	IQR
Track A	Top	0.050667	0.045333	0.074000	0.028667
Essix C+	Top	0.068833	0.049333	0.081833	0.032500
Erkodur A1	Top	0.059667	0.050000	0.076000	0.026000
Biolon	Top	0.064667	0.056500	0.073333	0.016833
Track A	Bottom	0.245333	0.081833	0.366333	0.284500
Essix C+	Bottom	0.464500	0.358667	0.598667	0.240000
Erkodur A1	Bottom	0.629333	0.480000	0.728000	0.248000
Biolon	Bottom	0.522167	0.415500	0.598000	0.182500

Suppl. Table S2. Descriptive statistics of surface roughness values (Ra) after thermocycling and brushing with three different toothbrushes on the top side of specimens

Brand	Description	MEDIAN	Q1	Q3	IQR
Track A	Ra after thermocycling	0.448333	0.374000	0.565667	0.191667
	Ra after brushing with CS 5960	0.877333	0.613000	1.119000	0.506000
	Ra after brushing with CS 3960	0.904000	0.629000	1.336667	0.707667
	Ra after brushing with CS 1560	1.038000	0.921000	1.516000	0.595000
Essix C+	Ra after thermocycling	0.438167	0.388333	0.557500	0.169167
	Ra after brushing with CS 5960	0.478500	0.366667	0.566667	0.200000
	Ra after brushing with CS 3960	0.786833	0.693000	0.868333	0.175333
	Ra after brushing with CS 1560	0.907833	0.665000	1.067000	0.402000
Erkodur A1	Ra after thermocycling	0.499750	0.337833	0.595500	0.257667
	Ra after brushing with CS 5960	0.473333	0.311333	0.551000	0.239667
	Ra after brushing with CS 3960	0.536167	0.437667	0.672667	0.235000
	Ra after brushing with CS 1560	0.744333	0.382000	1.112667	0.730667
Biolon	Ra after thermocycling	0.435333	0.348667	0.496833	0.148167
	Ra after brushing with CS 5960	0.776167	0.688333	0.821333	0.133000
	Ra after brushing with CS 3960	1.164500	0.840333	1.293333	0.453000
	Ra after brushing with CS 1560	1.444833	1.212000	1.872333	0.660333

Suppl. Table S3. Descriptive statistics of surface roughness change (ΔRa) after thermocycling and brushing with three different toothbrushes on the bottom side of specimens

Brand	Description	MEAN	STDEV	SD CI - 95%	SD CI + 95%
Track A	ΔRa after thermocycling	1.007181	0.363461	0.282487	0.509848
	ΔRa after brushing with CS 5960	-0.112889	0.533967	0.333306	1.309614
	ΔRa after brushing with CS 3960	0.025560	0.410678	0.256348	1.007234
	ΔRa after brushing with CS 1560	0.168111	0.525360	0.327934	1.288505
Essix C+	ΔRa after thermocycling	0.591410	0.351729	0.273368	0.493391
	ΔRa after brushing with CS 5960	-0.030611	0.223512	0.139518	0.548188
	ΔRa after brushing with CS 3960	-0.031444	0.425866	0.265829	1.044486
	ΔRa after brushing with CS 1560	0.437833	0.346017	0.215987	0.848646
Erkodur A1	ΔRa after thermocycling	0.439514	0.540164	0.417760	0.764522
	ΔRa after brushing with CS 5960	0.156667	0.207555	0.129558	0.509053
	ΔRa after brushing with CS 3960	0.034528	0.658495	0.411038	1.615035
	ΔRa after brushing with CS 1560	-0.051600	0.367234	0.220022	1.055267
Biolon	ΔRa after thermocycling	0.642262	0.298352	0.231883	0.418516
	ΔRa after brushing with CS 5960	0.045368	0.841922	0.525534	2.064909
	ΔRa after brushing with CS 3960	0.503327	0.613261	0.382803	1.504093
	ΔRa after brushing with CS 1560	1.094865	0.582062	0.363328	1.427573
